# Effect of feed supplementation with biosynthesized silver nanoparticles using leaf extract of *Morus indica* L. V1 on *Bombyx mori* L. (Lepidoptera: Bombycidae)

**DOI:** 10.1038/s41598-019-50906-6

**Published:** 2019-10-16

**Authors:** Sudip Some, Onur Bulut, Kinkar Biswas, Anoop Kumar, Anupam Roy, Ipsita Kumar Sen, Amitava Mandal, Octavio L. Franco, İkbal Agah İnce, Kartik Neog, Sandip Das, Sayantan Pradhan, Subhadeep Dutta, Debjoy Bhattacharjya, Soumen Saha, Pradeep K. Das Mohapatra, Anil Bhuimali, B. G. Unni, Ahmet Kati, Amit Kumar Mandal, M. Deniz Yilmaz, Ismail Ocsoy

**Affiliations:** 1 Chemical Biology Laboratory, Department of Sericulture, Raiganj University, Uttar Dinajpur, 733134 West Bengal India; 2 Department of Molecular Biology and Genetics, Faculty of Agriculture and Natural Sciences, Konya Food and Agriculture University, 42080 Konya, Turkey; 3 Department of Biological Sciences, Middle East Technical University, 06800 Ankara, Turkey; 4 Research and Development Center for Diagnostic Kits (KIT-ARGEM), Konya Food and Agriculture University, 42080 Konya, Turkey; 5 Laboratory of Organic Synthesis, Department of Chemistry, Raiganj University, Raiganj–733134, Uttar Dinajpur, West Bengal India; 6 ANMOL Laboratory, Department of Biotechnology, North Bengal University, Raja Ram Mohanpur, Siliguri, Darjeeling 734013 India; 7 Laboratory of Food Chemistry and Technology, Department of Chemical Engineering, Birla Institute of Technology, Mesra, Ranchi 835215 India; 8 Department of Science and Humanities, Sidhu Kanhu Birsa Polytechnic, Keshiary, 721133 West Bengal India; 9 Molecular Complexity Laboratory, Department of Chemistry, Raiganj University, Raiganj, 733 134, Uttar Dinajpur, West Bengal India; 10 S-INOVA Biotech, Post-Graduate Program in Biotechnology, Catholic University Dom Bosco, Campo Grande, Mato Grosso Do Sul Brazil; 11 Center of Proteomic and Biochemical Analysis, Post Graduate Program in Genomic Sciences and Biotechnology, Catholic University of Brasilia, Brasilia, Brazil; 12 Department of Medical Microbiology, School of Medicine, Acibadem Mehmet Ali Aydınlar University, 34752 Ataşehir, Istanbul Turkey; 13 Biotechnology Division, Central Muga Eri Research & Training Institute (CMER&TI), Central Silk Board, Ministry of Textiles: Govt. of India, Lahdoigarh-785700, Jorhat, Assam India; 14 Cytogenetics & Plant Biotechnology Research Unit, Department of Sericulture, Raiganj University, Uttar Dinajpur, 733134 India; 15 Department of Microbiology, Raiganj University, Uttar Dinajpur, 733134 West Bengal India; 16 Hon’ble Vice-Chancellor, Raiganj University, Uttar Dinajpur, 733134 West Bengal India; 17 Director Research, Assam Down town University, Sankar Madhav Path, Gandhi Nagar, Panikhaiti, Guwahati, 781026 Assam India; 18 Department of Biotechnology, Institution of Health Science, University of Health Science, Istanbul, 34668 Uskudar, Istanbul Turkey; 19 Department of Bioengineering, Faculty of Engineering and Architecture, Konya Food and Agriculture University, 42080 Konya, Turkey; 20 Department of Analytical Chemistry, Faculty of Pharmacy, Erciyes University, Kayseri, 38039 Turkey

**Keywords:** Nanoparticles, Nanoparticles

## Abstract

Herein, we report the synthesis of silver nanoparticles (AgNPs) by a green route using the aqueous leaf extract of *Morus indica* L. V1. The synthesized AgNPs exhibited maximum UV-Vis absorbance at 460 nm due to surface plasmon resonance. The average diameter (~54 nm) of AgNPs was measured from HR-TEM analysis. EDX spectra also supported the formation of AgNPs, and negative zeta potential value (−14 mV) suggested its stability. Moreover, a shift in the carbonyl stretching (from 1639 cm^−1^ to 1630 cm^−1^) was noted in the FT-IR spectra of leaf extract after AgNPs synthesis which confirm the role of natural products present in leaves for the conversion of silver ions to AgNPs. The four bright circular rings (111), (200), (220) and (311) observed in the selected area electron diffraction pattern are the characteristic reflections of face centered cubic crystalline silver. LC-MS/MS study revealed the presence of phytochemicals in the leaf extract which is responsible for the reduction of silver ions. MTT assay was performed to investigate the cytotoxicity of AgNPs against two human cell lines, namely HepG2 and WRL-68. The antibacterial study revealed that MIC value of the synthesized AgNPs was 80 µg/ml against *Escherichia coli* K12 and *Staphylococcus aureus* (MTCC 96). Finally, the synthesized AgNPs at 10 µg/ml dosages showed beneficial effects on the survivability, body weights of the *Bombyx mori* L. larvae, pupae, cocoons and shells weights via enhancing the feed efficacy.

## Introduction

Nanomaterials have drawn the keen interest of the researchers in this decade for their unique physico-chemical and biological properties with versatile applications in the field of agriculture, cosmetics, healthcare, medicine, industries, and packaging accessories^1–3^. There have been extensive research focusing on the synthesis of different metal and metal oxide nanoparticles such as silver nanoparticles (AgNPs), gold nanoparticles, zinc oxide nanoparticles through both conventional and non-conventional methods^4–6^. Among various nanomaterials, manufacture and application of Ag and AgNP-based materials have become a very active field in this cutting-edge technology for its excellent morphology, stability, and biophysical properties^7,8^. It has been extensively used in the biomedical field as antibacterial, antifungal, antiviral, anti-inflammatory, anti-angiogenic, and anti-cancer agents^9–13^.

Physical and chemical methods are the two popular synthetic routes for the fabrication of nanoparticles (NPs). The major drawbacks in chemical and physical methods of AgNPs preparation are that they are mostly expensive, time-consuming and involve use of toxic and hazardous chemicals, which constrain their applications in the biomedical and clinical field^14,15^. In order to overcome these difficulties and hurdles, biogenic synthesis of metallic nanoparticles has been established a new approach in green chemistry. In this process, biological extracts obtained from various organisms including plants, algae, microorganisms act as reducing as well as capping agents^16,17^. The main advantages of using biological entities for green synthesis of nanoparticles are that they are abundant, safe to handle and possess a variety of metabolites as reducing and capping agents^18^. Many biological resources especially the terrestrial and aquatic plants and algae are rich in secondary metabolites such as terpenoids, polyphenols, sugars, alkaloids, flavonoids, phenolic acids which play a pivotal in the bio-reduction silver ions and preventing the aggregation of the formed nanoparticles^19–28^. Extract of different plant parts like bark of *Pongamia pinnata*, leaves of *Azadirachta indica* and *Eriobotrya japonica*, and fruits of *Malus* (apple) have already been used for biofabrication of AgNPs^29–32^.

The plant *Morus indica* L. V1 which is more commonly known as mulberry under the family Moraceae has great importance in the sericulture field for completing the life cycle of the silkworm, *Bombyx Mori* L^33^. Previously, various species of *Morus* have been shown to have the antioxidant capacity and contain several polyphenol constituents such as rutin, isoquercitrin and astragalin^34,35^. The mulberry silkworm is an important and domestic insect and used for the production of the outstanding quality of silk. The worm is susceptible to various pathogenic attacks such as fungi, protozoan, viruses, and different types of Gram-positive and Gram-negative bacteria^36,37^. Flacheria is a disease of silkworms, caused by silkworms consuming mulberry leaves contaminated with several bacterial species such as *Bacillus subtilis*, *Streptococcus pneumoniae*, *Staphylococcus aureus*, *Escherichia coli*, *Pseudomonas fluorescence*, *Bacillus cereus* and *Klebsiella cloacae*^38^. In addition, “Sappe” is another bacterial disease of *B*. *mori* larvae, which played a pivotal role for excessive economic losses in the silk industry in Mysore of India. The bacterial species including *Aerobacter cloacae*, *Achromobacter superficialis*, *A*. *delmarvae*, *Pseudomonas boreopolis*, *P*. *ovalis*, *Escherichia freundii*, and *Staphylococcus albus* have been previously characterized and isolated from “Sappe” affected worms^39^.

The aim of the present study is the green synthesis of AgNPs using the leaf extract of *M*. *indica* L. V1 as both bio-reducing and capping agent. This study explores a faster, one-step, economic and eco-friendly synthetic route. To our knowledge, this is the in detail study on the synthesis of AgNPs using leaf extract of *M*. *indica* L. V1. Analytical techniques (UV-vis, DLS, FTIR, XRD, electron microscopy, and LC-MS/MS) were applied for characterization of the mulberry leaf extract and synthesized AgNPs. The cytotoxicity of AgNPs was evaluated against HepG2 and WRL-68 by MTT assay, while the antibacterial activity was determined against *Escherichia coli* K12 and *Staphylococcus aureus* (MTCC 96). Moreover, the effect of the synthesized AgNPs on larval, pupal, cocoons and shells weights of *Bombyx mori* L. was also evaluated.

## Materials and Methods

### Plant material, extraction procedure and synthesis of AgNPs

The plant material was prepared from fresh and healthy leaves of *M*. *indica* V1 which were first rinsed four times with deionized water to remove sand and debris, and then air-dried at ambient temperature. The aqueous extract was prepared by heating 15 g of finely ground leaves in 150 ml of deionized water at 90 °C approximately for 45 min, and filtered through Whatman filter paper No. 41 to remove any particles. The pale yellow clear solution was obtained and stored at 4–8 °C.

The biogenic synthesis of AgNPs was performed using silver nitrate (AgNO_3_) salt (Merck, USA) and *M*. *indica* V1 leaf extract as bio-reductant and capping agent. For this, 5 ml of leaf extract was added to 10 ml of 0.01 M AgNO_3_ solution and left at ambient temperature with continuous stirring at 200 rpm. The formation of AgNPs was confirmed by a color change from pale yellow to brown within 1 hr. The synthesized AgNPs were centrifuged at 12,000 rpm for 5 min and dispersed in deionized water for further studies.

### Characterization of synthesized AgNPs

#### UV-vis spectroscopy

The preliminary characterization of AgNPs was carried out using UV–Visible spectroscopy to monitor the reduction of Ag^+^ ions to the Ag^0^. The absorption spectra of the leaf extract and synthesized AgNPs were recorded with a UV-Vis spectrophotometer (Varian Inc., USA) in the range of 200–800 nm.

#### Nanoparticles size, polydispersity index (PDI) and zeta potential

Dynamic light scattering (Zetasizer Nano ZS90 ZEN3690, Malvern Instruments Ltd., UK) was used to measure the hydrodynamic diameter (d_h_), PDI and zeta potential of the synthesized AgNPs at 25 °C and at a scattering angle of 90° with He-Ne laser having emission wavelength of 632.8 nm.

#### Fourier-transform infrared spectroscopy (FTIR) analysis

FT-IR spectra of the as prepared aqueous leaf extract and synthesized AgNPs were studied in order to investigate the chemical compositions and functional groups using the FTIR spectrophotometer (Thermo Scientific Nicolet 380) equipped with a Helium Neon laser, deuterated triglycine sulfate detector and a KBr beam splitter in the wavelength range of 4000–400 cm^−1^ at room temperature. A small amount of liquid extract was taken in the glass capillary and added to the dry KBr powder, and then a pellet was prepared. This pellet was used for scanning the FTIR spectrum.

#### X-ray diffraction (XRD) analysis

XRD measurements were performed as described by Jain *et al*.^40^. The XRD pattern of synthesized AgNPs was recorded using Rigaku SmartLab (Japan), operating at 9 kW and CuKα radiation (λ = 1.54056 Å) in the range of 20° ≤ 2θ ≤ 80° at 40 keV. The lattice parameters were calculated by the PowderX software. The particle size (D) of the sample was calculated using the Scherrer’s equation as following; D = 0.9 λ/β cosθ, where λ, β, and θ represent the wavelength of X-ray, the broadening of the diffraction line measured as half of its maximum intensity in radians, and the Bragg’s diffraction angle, respectively. The particle size of the sample was estimated from the line width of the (111) XRD peak.

#### High resolution-transmission electron microscopy (HR-TEM) and energy-dispersive X-ray spectroscopy (EDX) analyses

The samples for HR-TEM analysis were prepared by carefully placing a single drop of aqueous synthesized AgNPs on a copper coated grid. TEM images were recorded using Jeol JEM-2100 electron microscope (Japan) operated at the voltage of 200 kV, at SAIF-NEHU, Shillong, India. In addition, EDX was also performed for the elemental analysis of the synthesized AgNPs.

#### Liquid chromatography–mass spectrometry (LC-MS) study

The aqueous leaf extract of *M*. *indica* V1 was also characterized by LC-MS/MS to investigate the phytochemical composition. The analysis was performed using a 2D-nanoACQUITY UPLC System equipped with a SYNAPT G2 mass spectrometry (Waters, USA). A positive mode of electrospray ionization (ESI) was employed. The source and desolvation temperatures were set as 100 °C and 350 °C, respectively. The rates of cone gas flow and desolvation gas flow were 50.0 L/hr and 700 L/hr, respectively. The identification of compounds in the extract was based on Flavor2 and NIST14 libraries as well as comparison of their retention indexes with previous reports.

### Cytotoxicity study

#### Maintenance of human cell cultures

HepG2 (human hepatocellular carcinoma cell line) and WRL-68 (hepatic fetal human epithelial cell line) were procured from National Centre for Cell Science (NCCS), Pune, India. Both cell lines were cultured in Dulbecco’s modified Eagle’s medium (DMEM) F-12 Ham supplemented with 10% fetal calf serum (FCS), 10 U/ml penicillin G and 100 µg/mL streptomycin in tissue culture dishes. The cells were maintained in a humidified incubator with 5% CO_2_ at 37 °C. When the cells reached approximately 80–90% confluency, disassociation was performed by trypsinizing the cells with 1X Trypsin-EDTA with prior to washing of cells with 1X PBS. The trypsin treated cells were incubated for 5 min, centrifuged for 5 min at 200 × g, and cell pellet was resuspended in fresh cell growth media. Equal number of cells (approximately 5 × 10^3^ cells) was seeded in each well of 96-well microplate, and the plate was incubated at 37 °C in a 5% CO_2_ incubator for minimum 24 h until the proper confluency was obtained. These cells were used for the cytotoxicity study.

#### Cytotoxic activity

In order to investigate cytotoxicity of synthesized AgNPs, MTT (3-[4,5-dimethylthiazole-2-yl]−2,5-diphenyltetrazolium bromide) assay, a colorimetric and indirect method for assessing the mitochondrial activity as a function of cell viability was performed according to the previous reports^41^. Briefly, the synthesized AgNPs at various concentrations (50 μg/ml, 100 μg/ml, 150 μg/ml, 200 μg/ml, 250 μg/ml) were treated with both cell lines in a a 96-well microplate, then the microplate was incubated for 24 h at 37 °C in a 5% CO_2_ incubator. After the incubation period, supernatants were replaced with 50 µl of MTT (1 mg/ml in 1X PBS), and incubated at 37 °C for 3 h. Then, 50 µl of isopropanol, formazan solubilizer, was added to each well and the plate was incubated for 5 min with shaking. The color developed was measured by recording the absorbance at 620 nm in a spectrophotometer. The percentage cell cytotoxicity was calculated as follows: % cell cytotoxicity = (A − B)/A × 100, where A is the absorbance of control (untreated) cells and B is the absorbance of the cells treated with varying concentrations of synthesized AgNPs.

#### Morphological study

The morphological changes induced by the synthesized AgNPs were also investigated using light microscopy. Briefly, HepG2 and WRL-68 cells were seeded in 35 mm polyvinyl coated cell culture plates and incubated at 37 °C in a CO_2_ incubator for 24 h. When the cells reach 80–90% confluency, the culture medium was replaced with fresh medium containing the synthesized AgNPs at various concentrations (50 µg/ml, 100 µg/ml, 150 µg/ml, 200 µg/ml and 250 µg/ml). After proper incubation, morphological changes of HepG2 and WR-68 cells were observed under a phase contrast inverted microscope (Olympus CK40-SLP, USA) at 200X magnification. The cell images were recorded by a digital camera (Olympus) attached to the microscope. The cells without any treatments served as the control.

### Antibacterial activity

The antibacterial activity of AgNPs synthesized using *M*. *indica* V1 leaf extract was evaluated against *Escherichia coli* K12 and *Staphylococcus aureus* (MTCC 96). Prior to the antibacterial assay, synthesized AgNPs were exposed to UV radiation for 1 h in order to remove any contaminants. The pure cultures of bacteria were subcultured on Muller Hinton (MH) agar plates. The overnight cultures of bacterial strains were inoculated to HM broth containing various concentrations of synthesized AgNPs (10 µg/ml, 20 µg/ml, 40 µg/ml and 80 µg/ml) and incubated at at 37 °C with vigorous shaking. The bacterial growth was monitored at different time intervals by measuring the optical densities at 600 nm (OD_600_) of the culture media. Minimum inhibitory concentration (MIC), the lowest AgNP concentration which prevents bacterial growth, was also calculated for the quantitative assessment. All the experiments were carried out in triplicate and mean values were reported. MH broth containing bacterial inoculums without AgNPs and containing AgNPs without any bacterial inoculums were used as negative and positive control, respectively.

### Effects of synthesized AgNPs on *Bombyx mori* L

The disease free layings (DFLs) of mulberry silkworm race, SK hybrids were collected from the Department of Sericulture, Govt. of Assam. The brushing and rearing of the silkworms was performed according to the standard procedures^42^. Mulberry variety V1 leaves obtained from the institute’s farm were used for the feeding of silkworms. The Mulberry leaves were first washed thoroughly with deionized water to clean the surface, and then air-dried. Selected concentrations of synthesized AgNPs (1 µg/ml, 10 µg/ml, 50 µg/ml and 100 µg/ml) were prepared in deionized water and spread evenly over the leaf surfaces using an atomizer when the larvae settled for the fourth moulting. Freshly ecdysis fifth instar larvae were then fed with the treated leaves taking three replications with 50 worms each. The treated leaves were first fed to the worms only once immediately after the worms moulted out, and remaining feedings were performed with untreated leaves. A control lot of larvae was fed with mulberry leaves alone was also maintained for comparison.

## Results and Discussion

### Phytochemical content and biosynthesis of AgNPs

Green chemistry offers a novel alternative over physical and chemical methods for synthesis of metallic nanoparticles by eliminating problems associated with these conventional methods and providing an economical and eco-friendly approach. Green synthesis is considered as an “bottom up approach” in which metal salts are reduced by the biological extract composed of various enzymes and secondary metabolites. Plant extracts have gained much attention due to non-toxic and safe metabolite content among various biological sources. The major plant bioactive compounds that mediate the reduction of silver ions include phenolic compounds, flavonoids, ketones, aldehydes, tannins, terpenoids and organic acids. Various mulberry species have been shown to be rich in these bioactive compounds and have high antioxidant capacity^34,43^. Due to these properties, mulberry extracts have been used for synthesis of different nanomaterials such as gold, silver and iron nanoparticles^5,44,45^.

**Figure 1:**
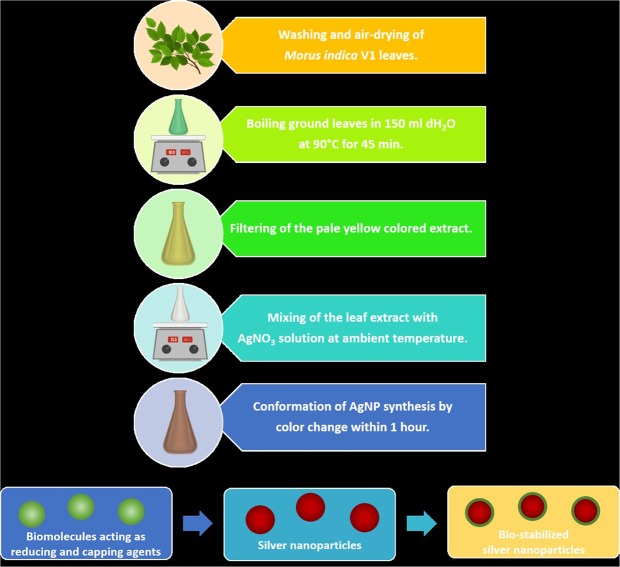
Schematic representation of the silver nanoparticle biosynthesis using the aqueous extract of *Morus indica* L. V1 as reducing and capping agents.

In the present study, leaves of mulberry *Morus indica* V1 were used for preparation of the biological extract, and its phytochemical content was characterized prior to the AgNP biosynthesis (Fig. 1). Under the used chromatographic and mass conditions, six peaks were detected for the mulberry leaf extract. Some compounds were identified by comparison of their LC retention time and ESI-MS spectrometric data with those of reference compounds while some peaks were assigned by comparing the ESI-MS/MS spectrometric data with the previous reports regarding components in *Morus alba* L^46–50^. The compounds identified by LC-QTOF/MS and HRMS spectra are represented in Supplementary Figs 1 and 2 respectively, and the results are summarized in Supplementary Table 1.

In *M*. *indica* V1, some bioactive substances namely isoquercetin, sophoraisoflavanone A, cyclomorusin, mangiferin xanthonoid, gallic acid, kazinol B and stigmasterol were identified and matched with previous reports. The first three compounds fall in flavonoids, a subclass of plant secondary metabolite, polyphenols. Mangiferin xanthonoid and gallic acid are phenolic compounds, one of the most widely occurring groups of phytochemicals^51^. Apart from these substances, kazinol B, a polyhydroxyflavan (benzopyran derivative), and stigmasterol, a plant steroid was also noted in *M*. *indica* V1 aqueous extract.

Previous reports have shown that phytochemicals such as phenolic compounds and flavonoids are directly associated with reduction of Ag^+^ ions into Ag^0^^40,52^. Furthermore, polyhydroxy compounds, especially flavonoids have a high tendency to chelate metal ions by forming stable complex through their multiple hydroxyl groups and the carbonyl moiety, therefore resulting in formation of silver nanoparticles^53^.

### Characterization of the synthesized AgNPs

#### UV-Vis spectroscopy

**Figure 2:**
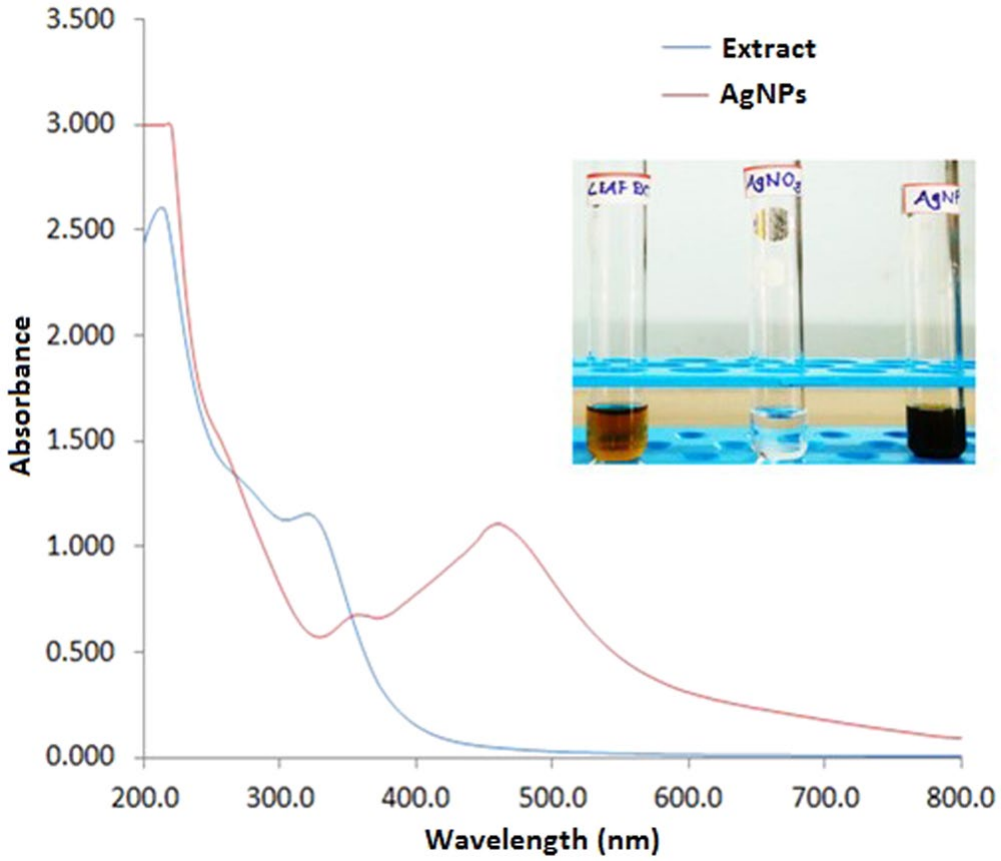
UV–visible spectra of the *Morus indica* L. V1 aqueous leaf extract and synthesized AgNPs. Figure inset showing visual colour changes of the leaf extract upon AgNO_3_ addition.

The aqueous mulberry extract mediated synthesis of AgNPs was initially monitored by UV-Vis spectroscopy (Fig. 2) and laser light scattering (Supplementary Fig. 3). Exposure of synthesized AgNPs to light leads to polarization of the free conduction electrons with respect to the much heavier ionic core of AgNPs, resulting in electron dipolar oscillation and appearance of a surface plasmon resonance band approximately at 460 nm. Absorption peak in the same wavelength range was not observed for the aqueous leaf extract solution used as a control.

#### Nanoparticles size, PDI and zeta potential

Hydrodynamic diameter (d_h_) is an important parameter that produces the morphological behavior of colloidal particles. The stability of nanoparticles is directly associated with the size distribution, which strongly depends on homogeneity of the medium. This was addressed by considering the PDI measured by DLS. The DLS measurements of synthesized AgNPs were performed in four distinct media, deionized water, PBS, LB broth and DMEM F-12, which illustrate its hydrodynamic diameter and polydispersity index (PDI), which correlates its potential stability in those medium. The synthesized AgNPs were found to have size distributions of 222.7 nm, 128.8 nm, 71.04 nm and 342 nm in deionized water, PBS, LB broth and DMEM F-12, respectively (Supplementary Fig. 4). The average d_h_ and PDI values of synthesized AgNPs in different medium are represent in Table 1. The nanoparticle size varied depending on the medium; as LB broth generated the smallest size distribution while DMEM F-12 yielded the largest distribution. Zeta potential of nanoparticles provides the evidence of nature and magnitude of surface charge which is associated with its physical stability. The synthesized AgNPs showed a zeta potential of −14.0 mV in deionized water, and PDI values in the range of 0.3 to 0.5, indicating low size variability and physico-chemical stability (Supplementary Fig. 5).

**Table 1:** Physical characterization of the synthesized AgNPs in various media at pH 7.2.

Deionized water		PBS		LB broth		DMEM F-12	
DLS (nm)	PDI	DLS (nm)	PDI	DLS (nm)	PDI	DLS (nm)	PDI
222.7 ± 22.26	0.535 ± 0.053	128.8 ± 6.022	0.415 ± 0.02	71.04 ± 1.07	0.32 ± 0.01	342 ± 0.9	0.4 ± 0.02

Note: Data are expressed as mean ± SD (n = 3).

#### FTIR and XRD analyses

**Figure 3:**
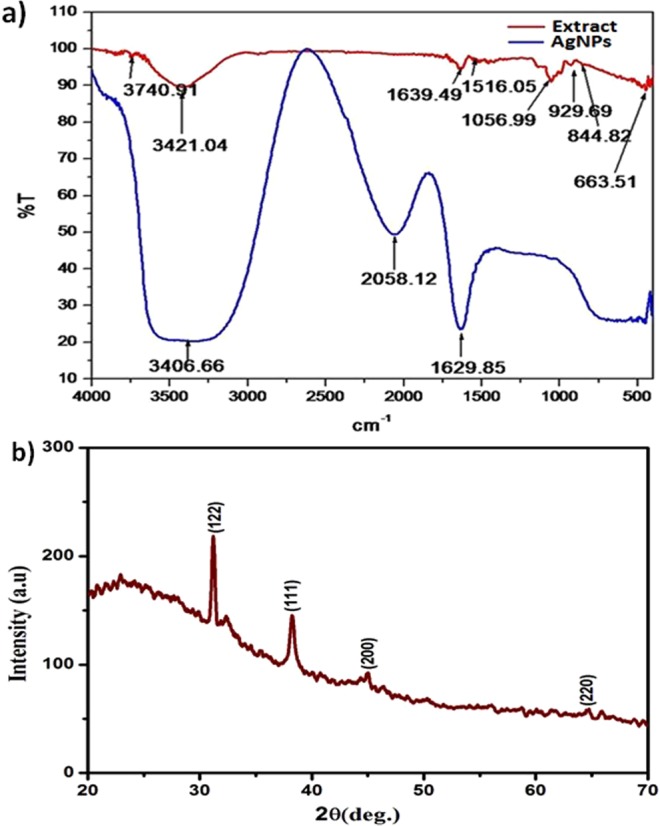
(**a**) FTIR spectra of the *Morus indica* L. V1 aqueous leaf extract and synthesized AgNPs; and (**b**) XRD pattern of the synthesized AgNPs.

The FTIR analysis was carried out to identify major functional groups present in the *Morus indica* V1 leaf extract, which are responsible for the synthesis of AgNPs. These functional groups present in the leaf extract might be responsible for the reduction of silver ions (Ag^+^) to silver nanoparticles (Ag^0^). The FTIR spectrum of mulberry leaf extract revealed the presence of sharp absorption peaks at 663, 1056, 1639 and 3421 cm^−1^ (Fig. 3a). The absorption peak at 1639 cm^−1^ was assigned to strong stretching vibrations of carbonyl group of *α*, *β* -unsaturated compounds. The broad peak at 3421 cm^−1^ indicated the presence of OH stretching in flavonoids, xanthonoids and phenolic compounds, while the peak at 1056 cm^−1^ appeared due to C‒O stretching^54,55^. The absorption pattern of the synthesized AgNPs showed the carbonyl stretching frequency at 1630 cm^−1^. The shifting of carbonyl stretching frequency from higher (in extract) to lower value (in AgNPs) is attributed due to the reduction of silver ions (Ag^+^) by the natural products present in leaves.

In XRD pattern (Fig. 3b), the presence of Braggs reflections arises due to (122), (111), (200) and (220) planes and agrees well with those reported for face center cubic (fcc) lattice structure of silver^40^. The XRD pattern clearly shows the crystalline nature of the silver nanoparticles.

#### HR-TEM and EDX analyses

**Figure 4:**
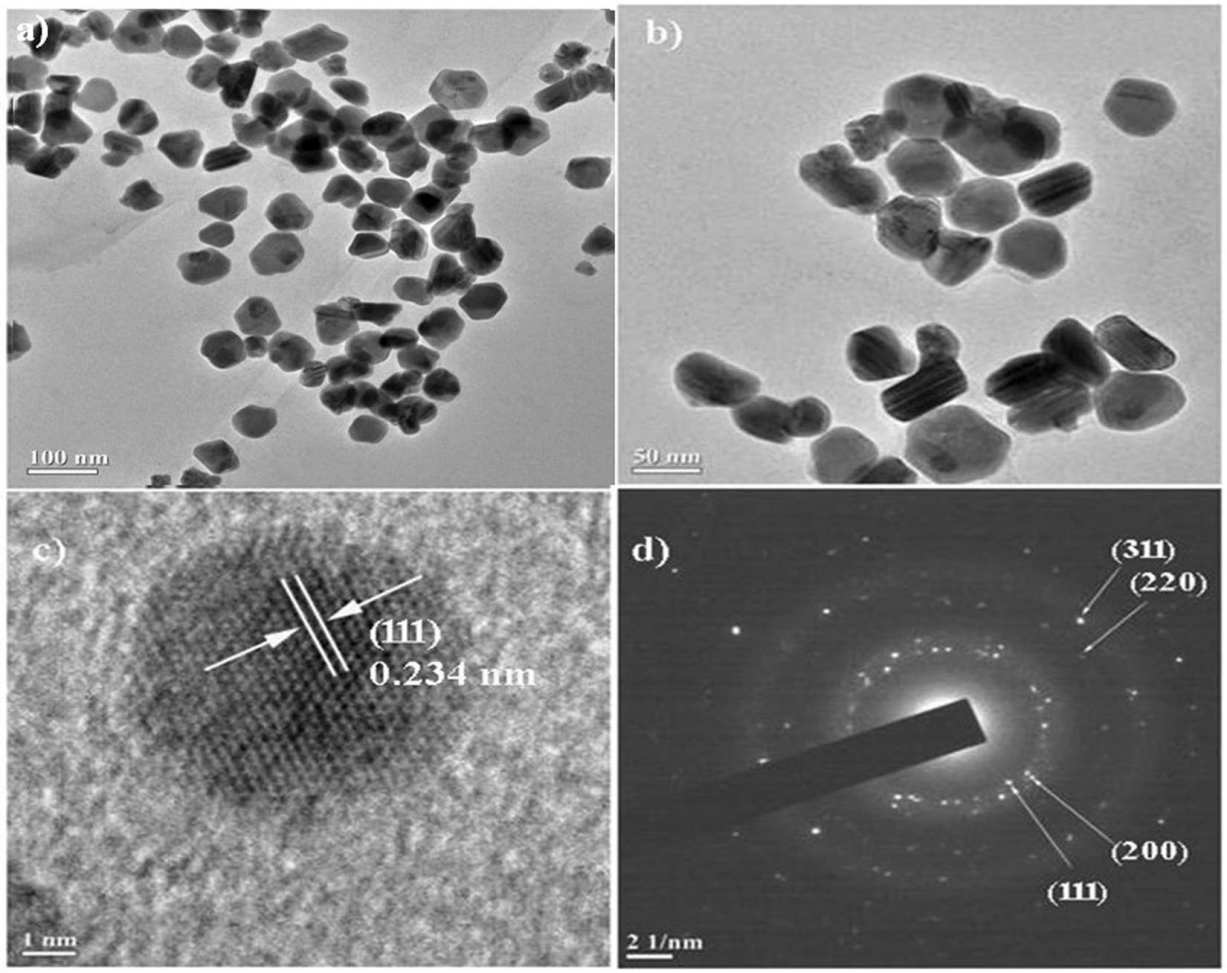
(**a**,**b**) HR-TEM images of the synthesized AgNPs (inset showing the particle size distribution); (**c**) lattice fringes of the synthesized AgNPs; (**d**) Selected area electron diffraction pattern of the face-centred cubic crystalline silver.

The HR-TEM was performed to visualize the size and morphology of the synthesized AgNPs. The TEM micrographs showed that the synthesized AgNPs were nearly quasi-spherical in shape with average particle size of ~54 nm, and well dispersed and scattered in nature (Fig. 4a,b). The visual analysis also showed the presence of a faint thin layer around the synthesized AgNPs, which confirms that biomolecules present in the leaf extract acted as a capping agent and also prevented aggregation of the nanoparticles. The capping of the synthesized AgNPs was further supported by the EDX analysis (Supplementary Fig. 6).

The lattice fringe with a distance of 0.234 nm shown in the HR-TEM image (Fig. 4c) further confirms the crystalline nature of the synthesized AgNPs. The four bright circular rings assigned to (111), (200), (220) and (311) observed in the selected area electron diffraction (SAED) pattern (Fig. 4d) are the characteristic reflections of face centered cubic crystalline silver^56^.

### Proposed mechanism for the synthesis of AgNPs

Despite various metallic nanoparticles have been synthesized using biological sources such as plants, microorganisms, algae and fungi, the exact mechanism of synthesis is still unknown. However, it has been proposed that the nanoparticle synthesis occurs in three main steps: (1) reduction of metal ions, (2) clustering, and (3) the nanoparticle formation^57^. Reactions take place in each of these steps directly pertain the temperature, pH, composition and concentration of the biological material, and metal salt concentration. In addition, the microbial reduction of nanoparticles by reductases and other equivalent reductants, and NADPH-mediated reduction of AgNO_3_ to silver nanoparticles were already reported in the literature^58^. The biological extracts containing naphthaquinones and anthraquinones moieties have sufficient redox potential for metal ion reduction and could act as electron shuttles^59^. According to the previous reports, flavonoids especially the -OH groups present in flavonoids are responsible for the reduction of silver ions. It has been proposed that hydrogen ions are released during the tautomeric transformation of enol form of flavonoids to keto form, resulting in the reduction of silver ions and synthesis of silver nanoparticles^21,40,53^.

**Figure 5:**
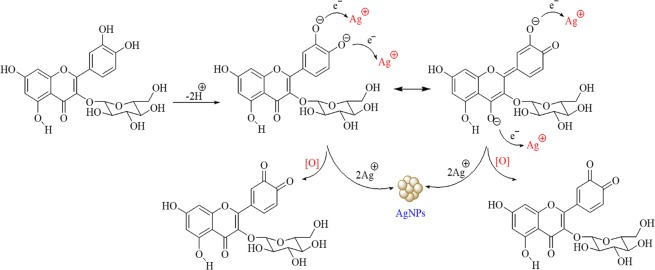
Illustration of the proposed mechanism for the synthesis of AgNPs by phytochemical mediated reduction of silver ions.

Different parts of mulberry species such as fruits and leaves have been shown to be rich in phytochemicals, particularly the phenolic compounds and flavonoids^15,60^. The leaf extract used in the present study contains a high amount of metabolites composed of aromatic rings having reactive -OH groups, which have been presumed to be acting as reducing and capping agents. The capping of synthesized AgNPs also observed in HR-TEM analysis might contribute to the stability of nanoparticles via preventing the agglomeration^61^. The proposed mechanism for the synthesis of AgNPs using the mulberry extract is summarized in Fig. 5. Briefly, AgNO_3_ molecules in the aqueous environment disassociate into silver ions (Ag^+^) and nitrate ions (NO_3_^−^). Upon the release of these two protons from flavonoid molecule, it leads to the reduction of two silver ions which cluster together resulting the formation of the silver nanoparticles.

### Cytotoxic activity

**Figure 6:**
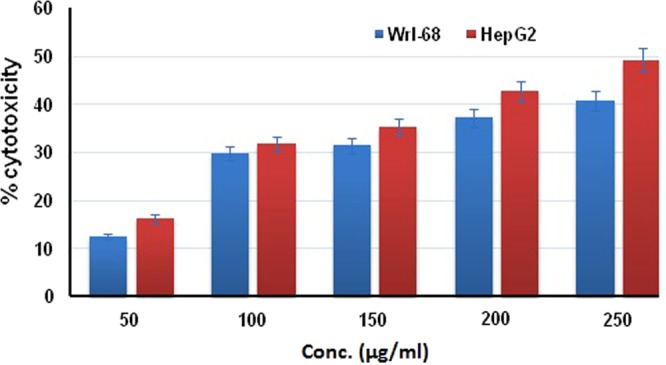
Dose-dependent cytotoxic activity of the synthesized AgNPs against human cell lines, HepG2 and WRL-68.

The cytotoxic effects of synthesized AgNPs against HepG2 and WRL-68 cell lines were evaluated by MTT assay. This assay is based on the reduction of the yellow aqueous solution of tetrazolium salt, 3-(4,5-dimethylthiazol-2-yl) to a violet blue/purple colored water insoluble dye compound, formazan by mitochondrial dehydrogenases present in metabolically active cells. Therefore, the amount of formazan is directly proportional to the number of viable cells^41^. The assay results showed the dose-dependent toxicity of the synthesized AgNPs towards both cell lines. With an increase in the concentration of synthesized AgNPs, decrease in cell viability was observed. The highest toxicity values of 41% and 49% were obtained at the highest nanoparticle concentration of 250 µg/ml in WRL-68 and HepG2 cells, respectively. Accordingly, the toxic effects of synthesized AgNPs gradually decreased to 37% and 43% at 200 µg/ml concentration, 32% and 35% at 150 µg/ml concentration, 30% and 32% at 100 µg/ml concentration, and finally declined to 12% and 16% at lowest concentration of 50 µg/ml, in WRL-68 and HepG2 cells, respectively (Fig. 6). The half-maximal inhibitory concentration (IC_50_) values were determined to be higher than 250 µg/ml. The cytotoxicity calculations were performed by comparing the treated cells with untreated control cells. At the same concentration, the synthesized AgNPs exhibited similar toxicity in both normal (WRL-68) and tumour (HepG2) cells.

The cytotoxic effects of biogenic AgNPs have been extensively studied. Several groups found that 50 µg/ml of AgNPs caused approximately %50 decrease in cell viability, which is higher than our results^62–64^. In the study of Selvan *et al*. (2018), AgNPs synthesized using different biological materials exhibited a dose-dependent toxic effect on both tumour and normal cells. IC_50_ values of biogenic AgNPs were in the range of 11–33 µg/ml in tumour cells, while IC_50_ value was found to be higher in the case of normal cells^65^. Interestingly, some research groups reported that biogenic AgNPs were more toxic than nanoparticles synthesized by chemical routes towards tumour cells, and biogenic AgNPs had no significant cytotoxic effect on normal cells^66,67^. In contrast, there have been reports indicating non-toxicity of biogenic AgNPs against various cell lines^68,69^.

Since many toxicological studies reporting different results have been published, it is difficult to make a certain conclusion about the toxicity of AgNPs. This difference presumably arises from the differences in biological materials used for the synthesis, nanoparticle size, shape, surface coating and cell type^70^. Therefore, the toxicity issue should be evaluated on a case by case basis. The synthesized AgNPs in this study did not exhibit severe toxicity, besides it did not have any effect at lower doses. The IC_50_ was determined to be more than 250 µg/ml in both cell lines, which is higher than many other AgNPs reported in the literature.

The cell morphology of HepG2 and WRL-68 cells after exposure to various concentrations of synthesized AgNPs was also evaluated by an optical microscope, and the images are shown in Supplementary Figs 7 and 8, respectively. As seen in the images, with the increase of concentration of AgNPs, the morphology of both cell lines gradually changed and distinct morphological changes indicating unhealthy cells were observed with respect to the untreated control cells. Particularly in HepG2 cells, the number of unhealthy spherical cells evidently increased in higher concentrations of AgNPs. However, the morphological changes in WRL-68 cells were not drastic as compared to HepG2 cells. Since the cell death mechanism varies depending on the cell type, the cellular response to an external agent might differ from cell to cell^71^.

### Antibacterial activity

**Figure 7:**
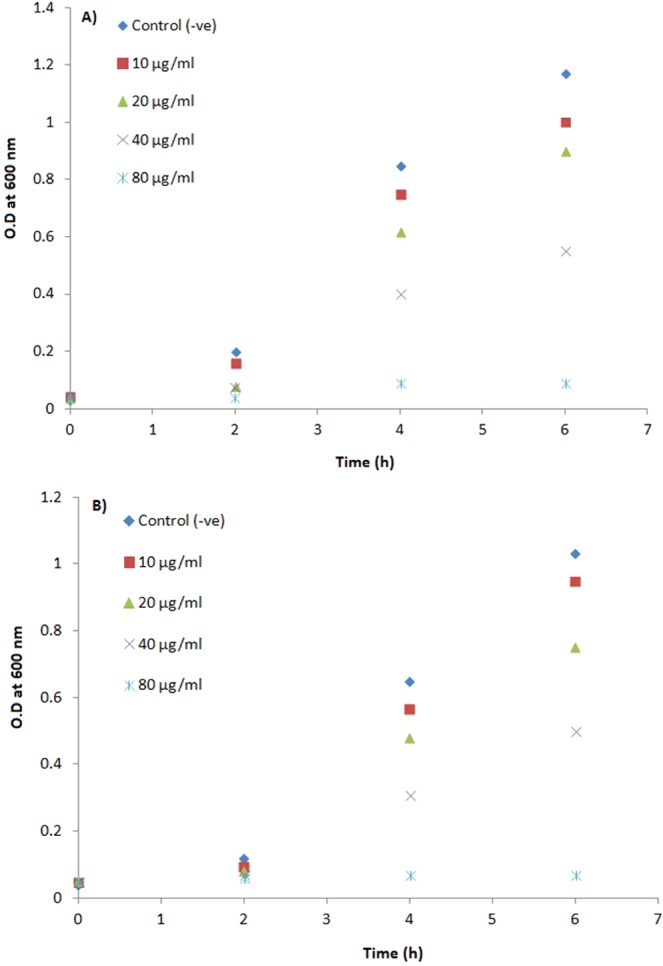
Growth curves of *Escherichia coli* K12 (**a**,**b**) *Staphylococcus aureus* (MTCC 96) in the presence of varying concentrations of synthesized AgNPs.

In this study, the antibacterial activity of synthesized AgNPs was tested against model systems of Gram-negative and Gram-positive bacteria, *Escherichia coli* K12 and *Staphylococcus aureus* (MTCC 96), respectively. The growth medium devoid of the synthesized AgNPs was used as a control, and growth profiles of treatments were compared to it. Control samples exhibited normal growth in MH broth, whereas the rates of bacterial growth decreased with the increase in AgNP concentrations in the case of both bacteria. The bacterial growth was completely arrested within 2 h in the presence of 80 µg/ml of AgNPs, and it was noted that no growth was observed even on overnight incubation. According to the dose-dependent activity of AgNPs, the bacterial growth was observed at lower concentrations (Fig. 7). Therefore, the concentration of 80 µg/ml was considered as MIC value for both *Escherichia coli* K12 and *Staphylococcus aureus*. During the incubation period, 40 µg/ml of AgNPs resulted in a 50% growth of test microorganisms as compared to the control, thereby designated as the median lethal dose (LD_50_).

The antimicrobial action of AgNPs is generally attributed to their effects on the target microbial cell, such as adhesion on the cell wall and membrane, triggering morphological changes by formation of pits, disruption of the cell integrity, impaired respiratory functions, inducing the oxidative stress by silver ion release, penetration inside the cell, and damaging the vital biomolecules including DNA, proteins and enzymes, which might individually or altogether result in the cell death. Size, shape, zeta-potential and capping agents are the major Physico-chemical characteristics which significantly affect the antimicrobial activity^17^. In a broad sense, smaller nanoparticles have higher antimicrobial activity due to the larger surface area to volume ratio^72^. Zeta-potential, on the other hand, is directly associated with antimicrobial activity since the interaction of nanoparticles with the cell membrane is based on electrostatic adhesion^73^. AgNPs synthesized in the present study have an average diameter of 71.04 ± 1.07 nm in the growth medium and a zeta-potential of –14.0 mV. The small size and less negative charge provide the nanoparticles with a higher interaction area and a definitive electrostatic attraction with the more negatively charged microbial cell membrane. These characteristics are mainly determined by the capping agents, the biomolecules present in the mulberry leaf extract.

### Effect of feed supplementation with biosynthesized silver nanoparticles on *Bombyx mori* L

Silkworms were fed with the synthesized AgNPs starting from the fifth instar, and their survivability, weights of larvae, cocoons and shells were measured and represented in Tables 2 and 3. The larvae seemed to live normally both in the presence and absence of AgNPs. Besides, the biosynthesized AgNPs reduced the larval mortality up to some extent. As seen in Table 2, the highest survivability of larvae (94.51%) was recorded in the group fed with mulberry leaves treated with 10 µg/mL of AgNPs, followed by 1 µg/ml (82.14%) and 50 µg/ml (78.33%) compared to that of control group (73.37%). On the other hand, the lowest survivability rate of 71.54% was recorded at a dose of 100 µg/ml. The larval stage plays a vital role in the growth and development of silkworms, thereby the larval weight is considered as an important parameter associated with the growth rate of larvae. Consistent with the larval survivability rates, the highest average larval weight of 3.721 ± 0.24 g was observed in the case of AgNPs at 10 µg/ml, whereas that of the control group was determined to be 3.422 ± 0.17 g. AgNPs at 1 µg/ml, 50 µg/ml, and 100 µg/ml concentrations resulted in 3.592 ± 0.21 g, 3.418 ± 0.23 g, and 3.227 ± 0.12 g of larval weights, respectively. Then, the alive silkworm larvae transformed into pupae and constructed the corresponding silkworm cocoons. The highest weight of pupae was 1.504 ± 0.32 g at 10 µg/ml concentration, whereas it was 1.322 ± 0.18 g in the control group. The pupa weight was decreased to 1.169 ± 0.15 at 100 µg/ml concentration, while other doses resulted in a slightly increase in the pupa weight. Conformably, treatment at 10 µg/ml resulted in the highest cocoon and shell weights, and doses at 50 and 100 µg/ml had a negative effect when compared to the control group.

**Table 2:** The effect of the synthesized AgNPs on the survivability of *Bombyx mori* L.

AgNPs concentration	Larval mortality (%)	Pupation rate (%)
1 µg/ml	17.50	82.14
10 µg/ml	9.64	94.51
50 µg/ml	20.14	78.33
100 µg/ml	25.87	71.54
Control	23.20	73.37

Note: Data are expressed as mean.

**Table 3:** The effect of the synthesized AgNPs on larval, pupal, cocoons and shells weights of *Bombyx mori* L.

AgNPs concentration	Mean weight of larvae (g)	Mean weight of pupae (g)	Mean weight of cocoons (g)	Mean weight of shells (g)
1 µg/ml	3.592 ± 0.21	1.420 ± 0.20	1.742 ± 0.22	0.326 ± 0.13
10 µg/ml	3.721 ± 0.24	1.504 ± 0.32	1.963 ± 0.30	0.467 ± 0.15
50 µg/ml	3.418 ± 0.23	1.363 ± 0.20	1.647 ± 0.21	0.279 ± 0.08
100 µg/ml	3.227 ± 0.12	1.169 ± 0.15	1.425 ± 0.22	0.257 ± 0.14
Control	3.422 ± 0.17	1.322 ± 0.18	1.633 ± 0.19	0.315 ± 0.14

Note: Data are expressed as mean ± SD.

**Figure 8:**
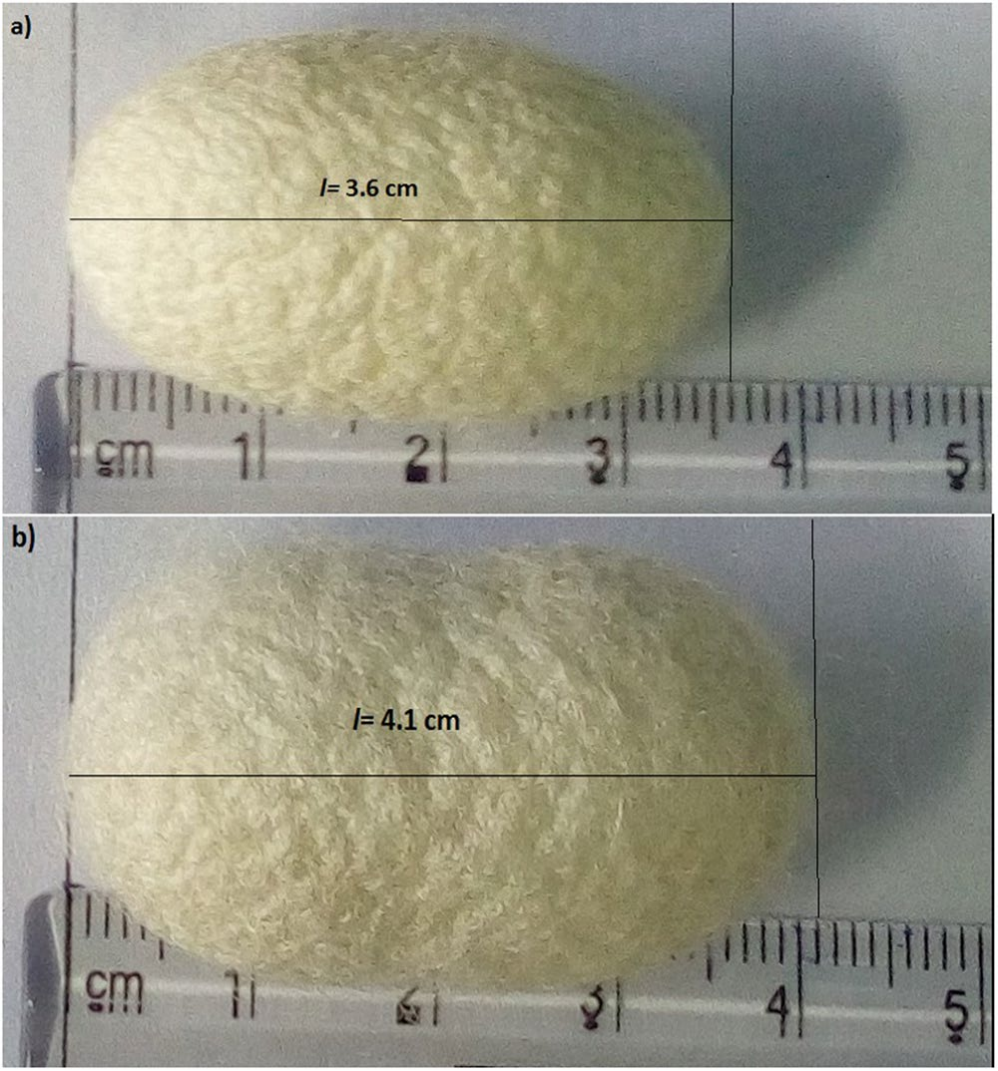
Effects of the synthesized AgNPs on the cocoon length; (a) untreated control, (b) AgNPs at 10 µg/ml concentration.

In summary, it is obvious that the synthesized AgNPs at 10 µg/ml concentration had a positive effect on the survivability, larval and pupal weights, and cocoon and shell weights by enhancing the feed efficiency. Moreover, the positive effects of the treatment with 10 µg/mL of AgNPs were visually observing the cocoon length which increased approximately by 13.9% in comparison to the control (Fig. 8). These results indicated that treatment of mulberry leaves with an adequate amount of the synthesized AgNPs can improve the larval survivability, weights of larvae, pupae, cocoons and shells; however negative effects were observed after the essential nanoparticle dose (>50 µg/ml), illustrating that effect of AgNPs on silkworm growth is dose-dependent.

Previously, Li *et al*. (2016) found that low concentrations of TiO_2_ NPs were effective for feed efficiency, weight gains, and cocoon mass, whereas higher concentrations had an inhibitory effect on the growth rate^74^. Similarly, Patil *et al*. (2017) demonstrated that the feeding of silkworms with green synthesized AuNPs did not only improve the cocoon and silk but also enhanced the amount of silk protein, fibroin^75^. In a separate study, it was showed that AgNPs at concentrations lower than 400 µg/ml promoted the growth and cocoon weight, but higher doses (≥800 µg/ml) of AgNPs resulted in silkworm death^76^. In contrast to these findings, Wu *et al*. (2017) reported that titanium, iron and copper NPs had no significant effect on silkworm weight, except for improving the mechanical properties of silk fibres^77^. It is estimated that approximately 2000 strains of *Bombyx mori* are present, each having different characteristics such as body weight, larval stage duration, cocoon weight, and other biological properties depending on the geographic origin^78^. In addition, interactions of nanoparticles with silkworm and the mechanism of these interactions are still unknown, but it is estimated to be arising from the physico-chemical properties and potent antimicrobial activities of NPs.

## Conclusions

Silver was treated as a noble metal in the comprehensive ancient Indian medical text, great “Charaka Samhita”. Since ancient times, silver has been used as an efficient therapeutic due to its beneficial properties. Recently, green synthesis of AgNPs has gained attention due to the use of biological resources, particularly the plant extracts. Metabolites present in these extracts serve as reducing and capping agents, and moreover determine the characteristics and behaviours of AgNPs. Nanoparticles obtained by green routes are considered as cost-effective, ecologically friendly, and non-toxic. With the increasing manufacture, widespread use and application areas of nanoparticles, safety issues for the biological applications have become more necessary. In the present report, AgNPs synthesized using the leaf extract of mulberry, *Morus indica* V1, exhibited high antibacterial activity against silkworm pathogens. Besides, the synthesized AgNPs improved silkworm survivability rates and increased larval, pupal and cocoon weights. Interestingly, these nanoparticles did not exhibit any significant toxic effect against both cell lines at concentrations used for antibacterial activity and beneficial effects on silkworms. As it is considered that other materials have been reported to have lower antibacterial activity and higher toxicity at the concentrations used in this study, the mulberry leaf extract mediated synthesized AgNPs have a valuable potential in biomedical applications.

## Supplementary information

Effect of feed supplementation with biosynthesized silver nanoparticles using leaf extract of Morus indica L. V1 on Bombyx mori L. (Lepidoptera: Bombycidae)
