# Supplementary material for: Effect of feed supplementation with biosynthesized silver nanoparticles using leaf extract of Morus indica L. V1 on Bombyx mori L. (Lepidoptera: Bombycidae)
Source: Sci Rep. 2019 Oct 16;9:14839. doi: 10.1038/s41598-019-50906-6 (PMC6795853; doi:10.1038/s41598-019-50906-6)
Supplement: Supplementary file 1 — Effect of feed supplementation with biosynthesized silver nanoparticles using leaf extract of Morus indica L. V1 on Bombyx mori L. (Lepidoptera: Bombycidae) [file 41598_2019_50906_MOESM1_ESM.docx]

**Effect of feed supplementation with biosynthesized silver nanoparticles using leaf extract of *Morus indica* L. V1 on *Bombyx mori* L. (Lepidoptera: Bombycidae)**

Mr. Sudip Some^1^, Mr. Onur Bulut^2,3,4^, Dr. Kinkar Biswas^5^, Dr. Anoop Kumar^6^, Dr. Anupam Roy^7^, Dr. Ipsita Kumar Sen^8^, Dr. Amitava Mandal^9^, Prof. Dr. Octavio L. Franco^10,11^, Prof. Dr. İkbal Agah İnce^12^, Dr. Kartik Neog^13^, Mr. Sandip Das^1^, Mr. Sayantan Pradhan^1^, Mr. Subhadeep Dutta^1^, Mr. Debjoy Bhattacharjya^14^, Dr. Soumen Saha^14^, Dr. Pradeep K. Das Mohapatra^15^, Prof. Anil Bhuimali^16^, Prof. B.G. Unni^17^, Dr. Ahmet Kati^12,18^, Dr. Amit Kumar Mandal^1,*^ Prof. Dr. M. Deniz Yilmaz^4,19*^, Dr. Ismail Ocsoy^20,*^

^1^Chemical Biology Laboratory, Department of Sericulture, Raiganj University, Uttar Dinajpur-733134, West Bengal, India

^2^Department of Molecular Biology and Genetics, Faculty of Agriculture and Natural Sciences, Konya Food and Agriculture University, 42080 Konya, Turkey

^3^Department of Biological Sciences, Middle East Technical University, 06800 Ankara, Turkey

^4^Research and Development Center for Diagnostic Kits (KIT-ARGEM), Konya Food and Agriculture University, 42080 Konya, Turkey

^5^Laboratory of Organic Synthesis, Department of Chemistry, Raiganj University, Raiganj–733134, Uttar Dinajpur, West Bengal, India

^6^ANMOL Laboratory, Department of Biotechnology, North Bengal University, Raja Ram Mohanpur, Siliguri, Darjeeling-734013, India

^7^Laboratory of Food Chemistry and Technology, Department of Chemical Engineering, Birla Institute of Technology, Mesra, Ranchi-835215, India

^8^Department of Science and Humanities, Sidhu Kanhu Birsa Polytechnic, Keshiary-721133, West Bengal, India

^9^Molecular Complexity Laboratory, Department of Chemistry, Raiganj University, Raiganj–733 134, Uttar Dinajpur, West Bengal, India

^10^S-INOVA Biotech, Post-Graduate Program in Biotechnology, Catholic University Dom Bosco, Campo Grande, Mato Grosso Do Sul, Brazil

^11^Center of Proteomic and Biochemical Analysis, Post Graduate Program in Genomic Sciences and Biotechnology, Catholic University of Brasilia, Brasilia, Brazil

^12^Department of Medical Microbiology, School of Medicine, Acibadem Mehmet Ali Aydinlar University, 34752 Ataşehir, Istanbul, Turkey

^13^Biotechnology Division, Central Muga Eri Research & Training Institute (CMER&TI), Central Silk Board, Ministry of Textiles: Govt. of India, Lahdoigarh-785700, Jorhat, Assam, India

^14^Cytogenetics & Plant Biotechnology Research Unit, Department of Sericulture, Raiganj University, Uttar Dinajpur-733134, India

^15^Department of Microbiology, Raiganj University, Uttar Dinajpur-733134, West Bengal, India

^16^Hon’ble Vice-Chancellor, Raiganj University, Uttar Dinajpur-733134, West Bengal, India

^17^Director Research, Assam Down town University, Sankar Madhav Path, Gandhi Nagar, Panikhaiti, Guwahati  781026, Assam, India

^18^Department of Detergent and Chemical Technologies, Hayat Kimya Research and Development Center, Kocaeli, 41250, Turkey

^19^Department of Bioengineering, Faculty of Engineering and Architecture, Konya Food and Agriculture University, 42080 Konya, Turkey

^20^Department of Analytical Chemistry, Faculty of Pharmacy, Erciyes University, Kayseri, 38039, Turkey

**Supplementary Table 1:** Spectrometric data of compounds found in Morus indica L. V1.

| **Sl No.** | **M (m/z) Cal.** | **M (m/z) found** | **Formula** | **Compound structure** | **Compound name** | **Ref** |
| --- | --- | --- | --- | --- | --- | --- |
| 1 | 464.0955 | 464.2910 | C_21_H_20_O_12_ |  | Isoquercetin | 36 |
| 2 | 170.0215 | 170.8545 | C_7_H_6_O_5_ |  | Gallic acid | 37 |
| 3 | 392.1988 | 392.1861 | C_25_H_28_O_4_ |  | Kazinol B | 38 |
| 4 | 370.1416 | 370.1861 | C_21_H_22_O_6_ |  | Sophoraisoflavanone A | 38 |
| 5 | 418.1416 | 418.1799 | C_25_H_22_O_6_ |  | Cyclomorusin | 38 |
| 6 | 422.0849 | 422.2616 | C_19_H_18_O_11_ |  | Mangiferin xanthonoid | 39 |
| 7 | 412.3705 | 412.1508 | C_29_H_48_O |  | Stigmasterol | 40 |


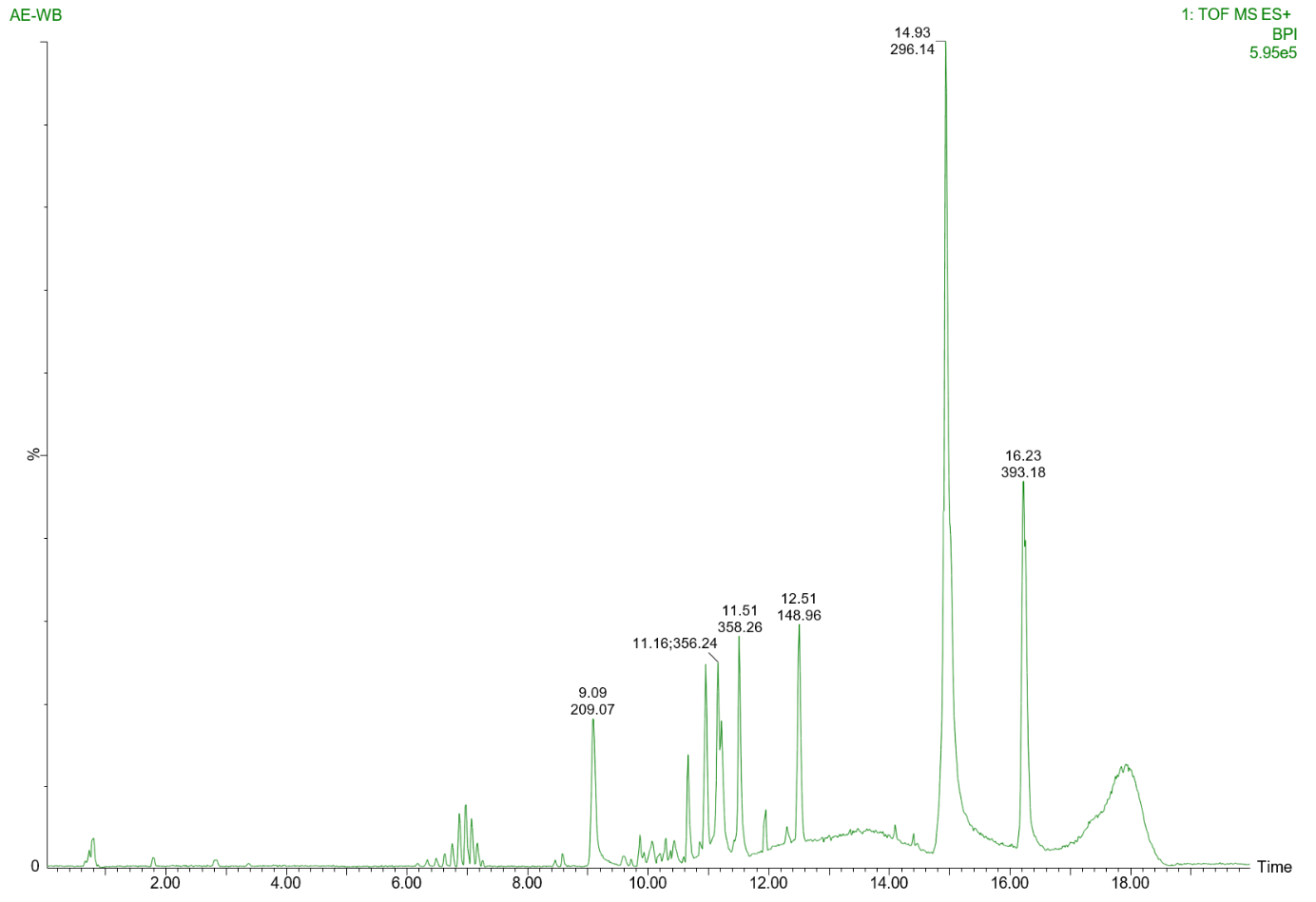


**Supplementary Figure 1:** Various components in the aqueous mulberry leaf extract identified by LC-QTOF/MS.


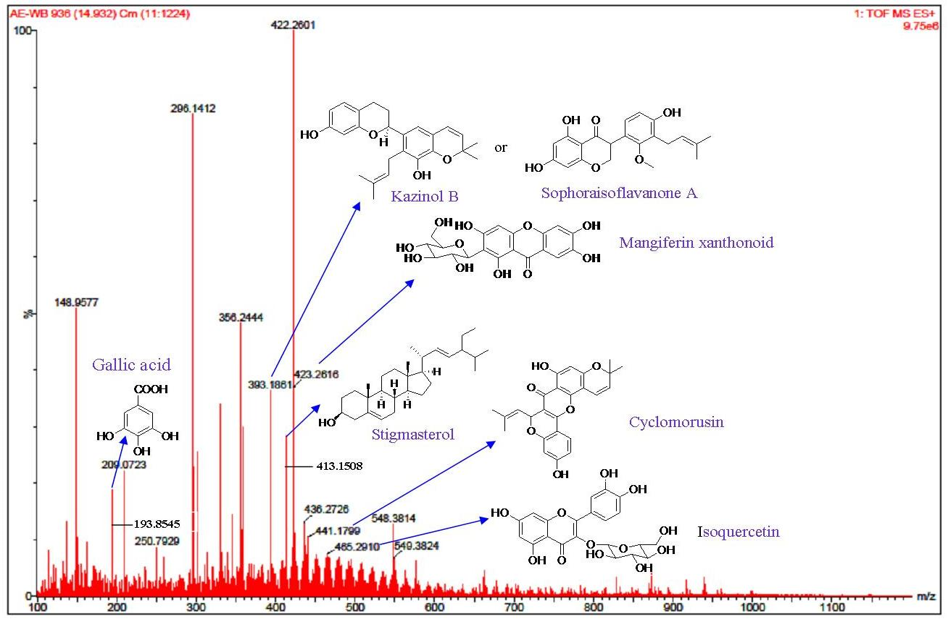


**Supplementary Figure 2:** Various peaks of the aqueous mulberry leaf extract assigned from HRMS spectra and corresponding compounds.

**
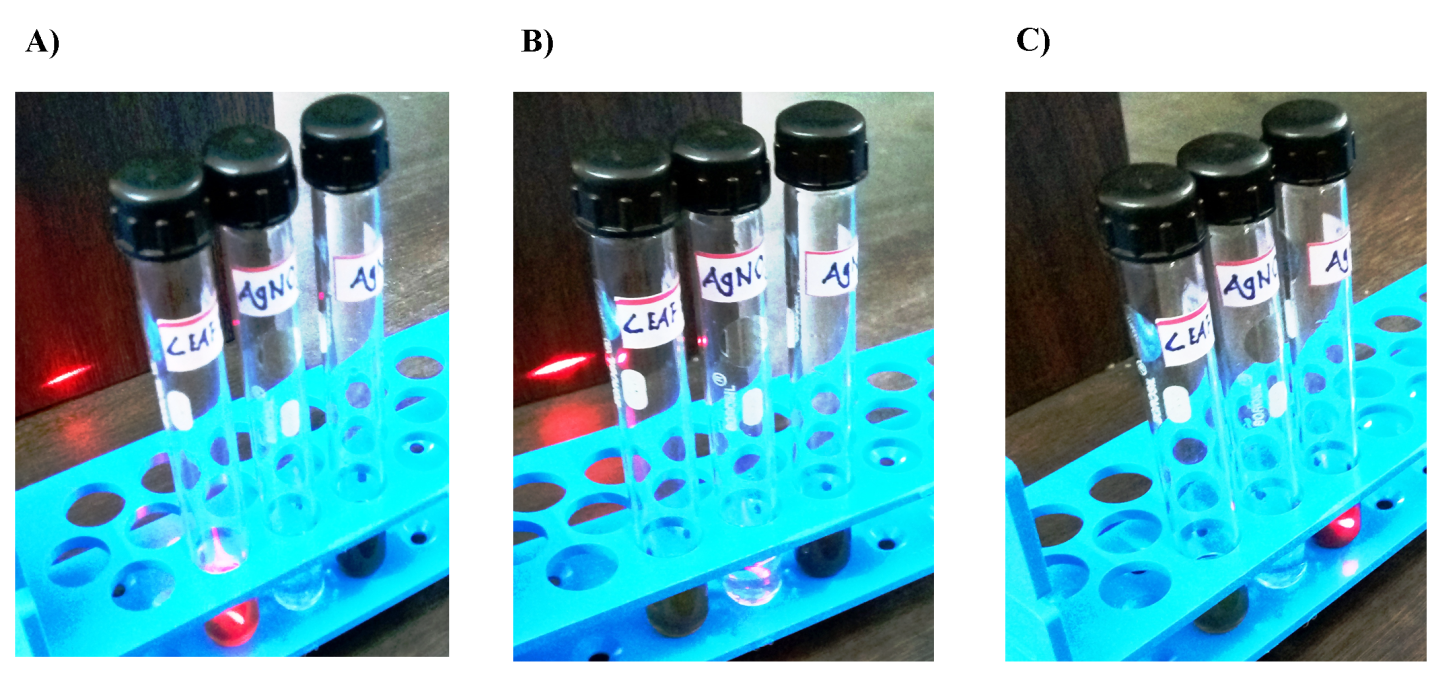
**

**Supplementary Figure 3:** Confirmation of the AgNP formation via laser light scattering: Laser light is passing through Morus indica V1 leaf extract (A), and 0.01 M AgNO_3_ solution (B). Laser light is scattered by AgNPs due to Tyndall scattering which confirms the formation of AgNPs (C).


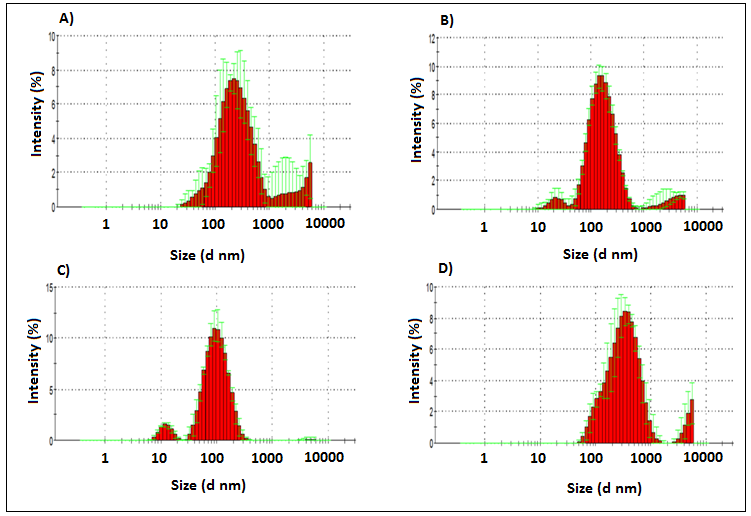


**Supplementary Figure 4:** Dynamic light scattering of the synthesized AgNPs in various media at pH 7.2: (A) Deionized water; (B) PBS; (C) LB broth, and D) DMEM F-12.

*
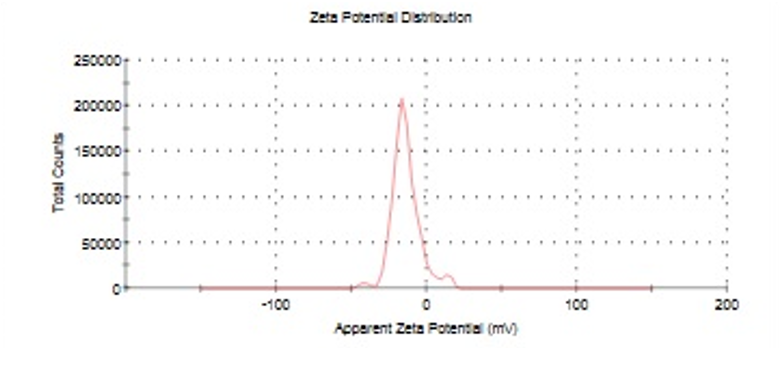
*

**Supplementary Figure 5:** Zeta potential distribution of the synthesized AgNPs.

**
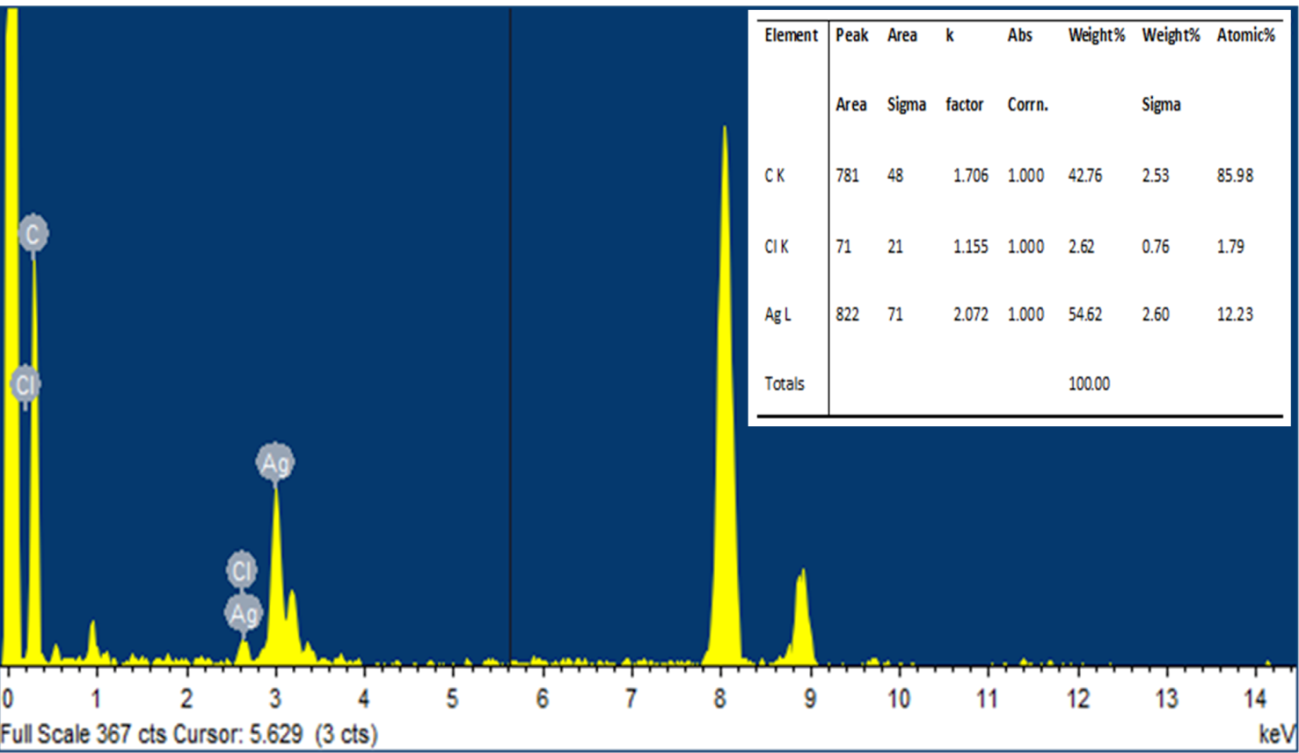
**

**Supplementary Figure 6:** EDX spectrum of the synthesized AgNPs.


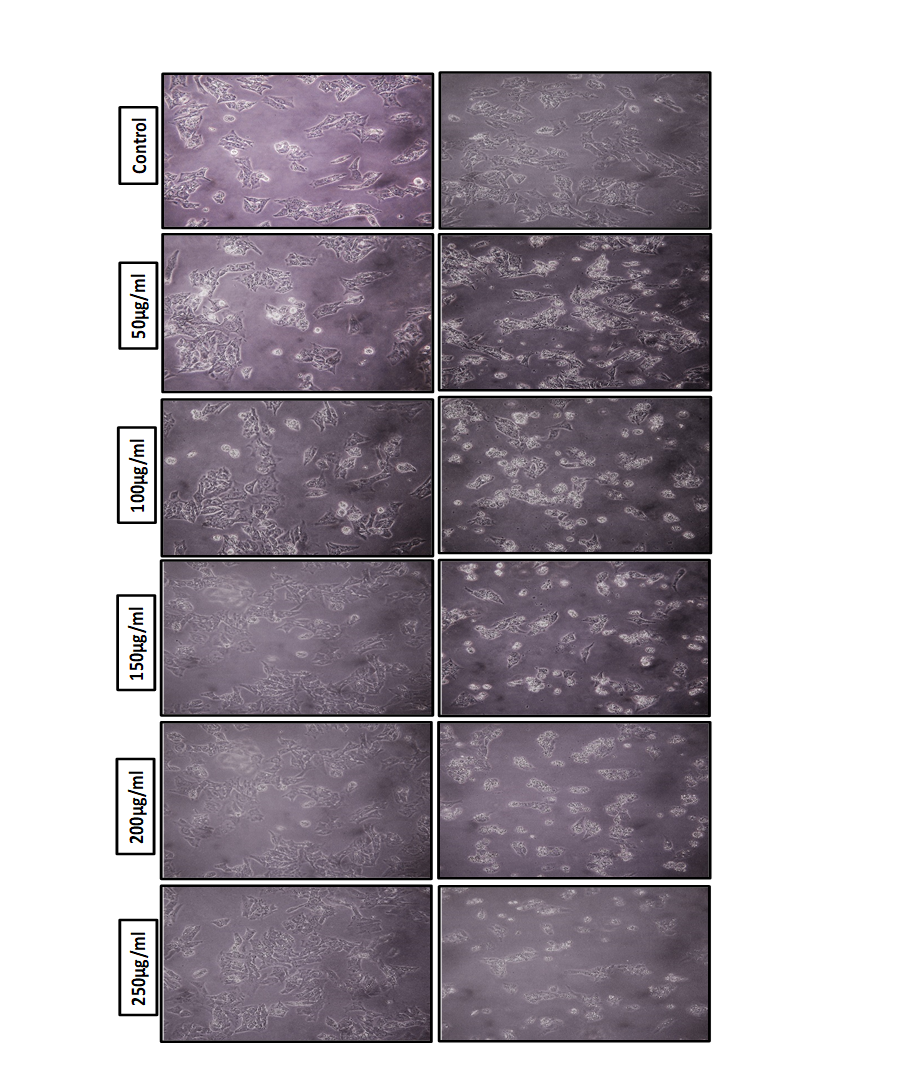


**Supplementary Figure 7:** The microscope images of HepG2 cells exposed to the synthesized AgNPs for 24 h.


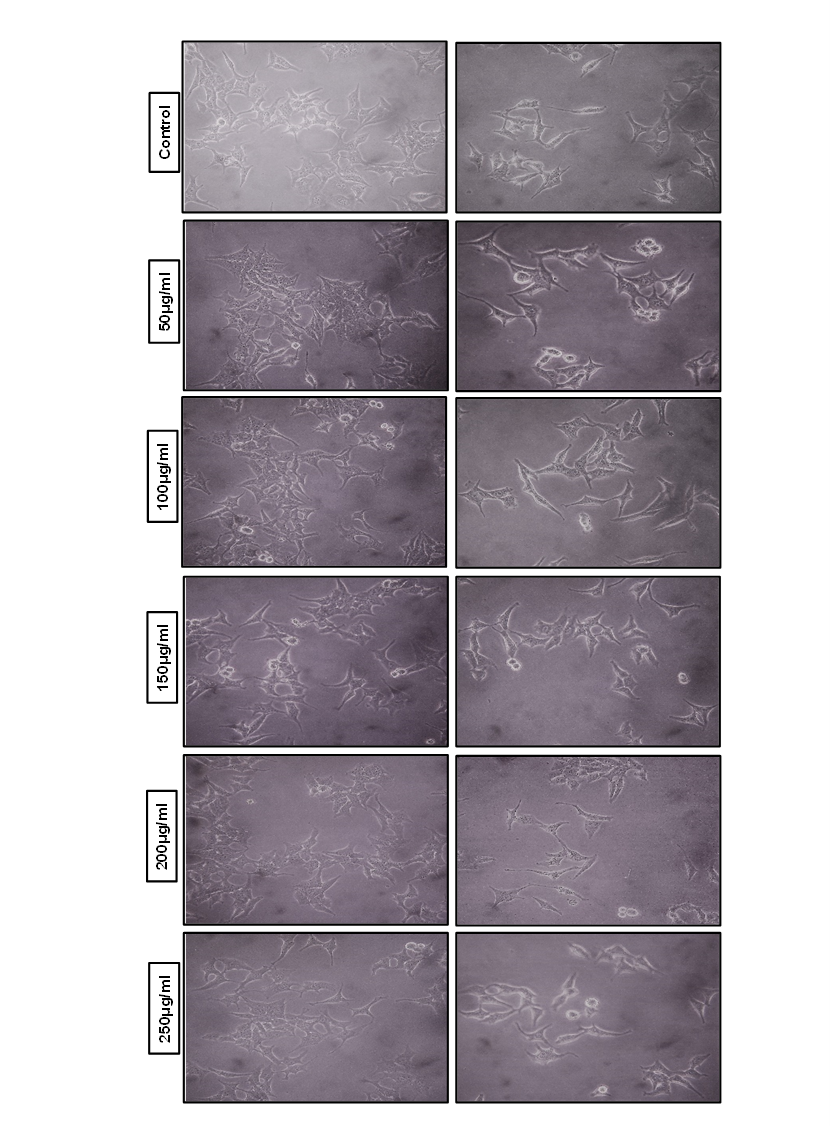


**Supplementary Figure 8:** The microscope images of WRL-68 cells exposed to the synthesized AgNPs for 24 h.
